# 754 A Longitudinal Study of Compression Therapy in Pediatric Burn Patients

**DOI:** 10.1093/jbcr/irad045.229

**Published:** 2023-05-15

**Authors:** Sinh Tran, Syra Han, Maria Saade Daoud, Deepak Gupta

**Affiliations:** Santa Clara Valley Medical Center, San Jose, California; Santa Clara Valley Medical Center, San Jose, California; Santa Clara Valley Medical Center, San Jose, California; Santa Clara Valley Medical Center, San Jose, California; Santa Clara Valley Medical Center, San Jose, California; Santa Clara Valley Medical Center, San Jose, California; Santa Clara Valley Medical Center, San Jose, California; Santa Clara Valley Medical Center, San Jose, California; Santa Clara Valley Medical Center, San Jose, California; Santa Clara Valley Medical Center, San Jose, California; Santa Clara Valley Medical Center, San Jose, California; Santa Clara Valley Medical Center, San Jose, California; Santa Clara Valley Medical Center, San Jose, California; Santa Clara Valley Medical Center, San Jose, California; Santa Clara Valley Medical Center, San Jose, California; Santa Clara Valley Medical Center, San Jose, California

## Abstract

**Introduction:**

Compression Therapy is crucial to managing and preventing hypertrophic scarring. The pediatric population can be especially at risk for hypertrophic scarring due to rapid growth periods and/or discomfort which interferes with compliance. Our previous practice was for pediatric patients to be scheduled every 6 -12 months like the adult burn population. To address this issue, we hypothesized pediatric patients would benefit from more frequent follow up visits. We arbitrarily determined to follow our pediatric patients every 3-4 months for garment remeasuring to improve proper garment fit, efficacy and compliance.

**Methods:**

A longitudinal study was conducted to determine whether 3–4 month interval check-ups would improve compliance of garment wearing and presumably efficacy of overall management of hypertrophic scarring. Data was collected between June 2020 – December 2021 (1 ½ years). The data was manually retrieved from our Electronic Medical Record.

**Results:**

Prior to June 2021, children were routinely scheduled in the Burn Clinic at intervals between six and twelve months for scar checks (same as adults). Children were not able to participate in long term compression therapy for the entire interval for several reasons including growth, size changes, garment failure, expired insurance authorization, and possibly lack of knowledge.

We decided to schedule consistent follow-up of pediatric patients every 3-4 months, to ensure consistent remeasuring of garments was completed.

Pre intervention results noted that only 35% of patients had a 2^nd^ garment remeasured as compared to our post intervention results which showed that 88% of patients successfully had a 2^nd^ garment remeasured.

**Conclusions:**

Our data suggests that short interval follow up with pediatric burn patients for scar management has led to improved rates of compression therapy compliance and presumably efficacy.

**Applicability of Research to Practice:**

We have continued the practice of short term follow up in pediatric burn patients for scar management and our data has also provided opportunities to reinforce education with patients and their families. We have found that with better understanding, our patients and families are motivated to seek out and comply with longitudinal care.

